# GFH312 Reduces Neuronal Death by Inhibiting RIPK1‐Mediated Apoptosis and Necroptosis After Ischemic Stroke

**DOI:** 10.1002/cns.71053

**Published:** 2026-10-07

**Authors:** Ze Liu, Shengju Wu, Qian Suo, Chang Liu, Rubing Shi, Jing Ye, Haroon Khan, Jing‐Yang Zhang, Yaohui Tang, Zhijun Zhang, Wanlu Li, Fu‐Sheng Zhou, Guo‐Yuan Yang

**Affiliations:** ^1^ School of Biomedical Engineering Shanghai Jiao Tong University Shanghai China; ^2^ GenFleet Therapeutics Inc. Shanghai China

**Keywords:** apoptosis, GFH312, necroptosis, RIPK1

## Abstract

**Aims:**

To investigate the role of the RIPK1 inhibitor GFH312 in ischemic stroke and identify its potential mechanisms.

**Methods:**

GFH312 was administered to adult mice in an ischemic stroke model. To ascertain the effectiveness of treatment, neurobehavioral tests and assessments of brain damage were carried out. Brain tissues were examined using immunofluorescence as well as western blot and real‐time quantitative PCR at various intervals following ischemia to evaluate inflammatory gene expression and signaling pathways linked to apoptosis and necroptosis.

**Results:**

Temporal analysis revealed that necroptosis and apoptosis were rapidly activated following ischemia, peaking at 1–3 days. GFH312 significantly improved neurological function and reduced brain injury in stroke mice. Mechanistically, GFH312 markedly inhibited apoptosis and necroptosis via suppression of the RIPK1‐related signaling pathway (RIPK1‐RIPK3‐MLKL). In addition, GFH312 greatly decreased inflammatory gene expression in the brain.

**Conclusion:**

Apoptosis and necrotic apoptosis occur primarily during the acute poststroke phase. GFH312 exerts neuroprotective effects after ischemic stroke, which are associated with suppression of RIPK1‐related apoptosis, necroptosis, and neuroinflammation.

## Graphical abstract

GFH312 alleviates ischemic stroke injury by targeting RIPK1 and limiting downstream necroptotic, apoptotic, and inflammatory responses. This study highlights the therapeutic potential of RIPK1 inhibition in stroke.

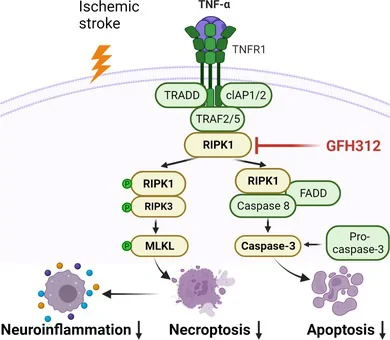

## 1 Introduction

Ischemic stroke ranks among the top causes of mortality and disability worldwide, caused by a temporary or permanent suspension or decrease of local cerebral blood flow [1, 2, 3]. Numerous types of cell death and neuroinflammation have been shown to be important factors in the pathogenesis of ischemic stroke [4]. Among them, it has been determined that receptor‐interacting serine/threonine‐protein kinase 1 (RIPK1) is a crucial mediator controlling inflammatory signaling pathways, necroptosis, and apoptosis [5]. However, there are currently no therapeutically licensed RIPK1 inhibitors, and the exact time behind the cell death activation in the brain after ischemic stroke remains unclear.

In the postischemic stroke setting, tumor necrosis factor‐alpha (TNF‐α) mediated signaling activates RIPK1 at an early stage, while caspase inhibition promotes the recruitment of RIPK3 and mixed lineage kinase domain‐like protein (MLKL) to RIPK1, resulting in the formation of amyloid‐like “necrosome” complex and ultimately inducing necroptosis [6, 7]. In RIPK1‐dependent apoptosis (RDA), RIPK1 assembles a signaling complex with FADD and caspase‐8, which then activates downstream caspases such as caspase‐3 to carry out apoptosis [5, 8]. Moreover, RIPK1 amplifies inflammatory responses by facilitating the production and secretion of key pro‐inflammatory cytokines such as TNF‐α, interleukin‐1 beta (IL‐1β), and interleukin‐1 alpha (IL‐1α) [9, 10, 11, 12]. Prior investigations have indicated that inhibition or knockdown of RIPK1 can protect against ischemic brain injury by attenuating infarct size and enhancing functional neurological recovery after stroke, thereby supporting its therapeutic potential [13, 14, 15].

The kinase structure of RIPK1 contains a hydrophobic pocket that facilitates binding with small‐molecule inhibitors, making it an ideal target for the development of specific drugs [16]. However, although RIPK1 has been recognized as a therapeutic target with great potential, the development of RIPK1‐targeted drugs is still at an early stage. Several RIPK1 inhibitors have entered clinical trials; no drug has yet been approved for clinical use, especially in ischemic stroke [17, 18]. GFH312 is a novel RIPK1 inhibitor and the first RIPK1 drug authorized for clinical trials in China. It has been evaluated in phase I/II clinical trials (NCT0467671 and NCT05618691) and has shown good safety and application prospects in other diseases. However, its therapeutic effects and mechanisms in ischemic stroke have not yet been verified.

Therefore, this study intended to assess the neuroprotective effects and underlying mechanisms of GFH312 in ischemic stroke. This work constructed temporary middle cerebral artery occlusion (tMCAO) and observed the dynamic changes of apoptosis and necroptosis after ischemic stroke. Subsequently, we comprehensively assessed the effects of GFH312 on infarct volume and neurological recovery and investigated its regulatory effects on apoptosis, necroptosis (RIPK1–RIPK3–MLKL pathway), and neuroinflammation. This research presents novel experimental evidence for the utilization of RIPK1 inhibitors in ischemic stroke and suggests possible support for their clinical application.

## 2 Methods

### 2.1 tMCAO Model

Adult ICR mice (25–30 g, 6–8 weeks; JSJ Company) were utilized for the investigations. The tMCAO procedure was conducted on the mice as previously documented [19]. For anesthesia, mice received isoflurane via an anesthesia machine (RWD, Shenzhen, China). A home‐made 6‐0 silicon‐coated suture was introduced to the middle cerebral artery. The suture remained in situ for 90 min to facilitate occlusion and was subsequently removed to reinstate blood flow. Animals in the sham group were subjected to the same operation, except that the suture was not advanced or withdrawn.

The animal study design is summarized in Figure 2a. Overall, 106 adult ICR mice were enrolled in the study, with 86 mice undergoing 90 min of tMCAO. GFH312, with a purity of 96.81% and dissolved in 0.5% carboxymethyl cellulose/0.2% Tween 80, was obtained from Shanghai Genfleet Company (Shanghai, China). The medication was provided to ischemic mice 15 min prior to reperfusion and subsequently twice daily for 3 days at 10 mg/kg by oral administration. A separate group of mice received the vehicle control.

### 2.2 Brain Injury Assessment

On the third day after tMCAO, mice were euthanized and evaluated for cerebral infarct and edema area using a previously described method [20]. After cardiac infusion of cold PBS, the mouse brain was promptly excised and rapidly frozen in prechilled isopentane (Macklin, Shanghai, China) at −80°C. The brains were embedded in OCT and sectioned into a series of 25‐μm slices.

Brain sections separated by 250 μm were stained using nissl staining solution (Beyotime, Shanghai, China). The dimensions of the ipsilateral healthy area, ipsilateral hemisphere, and contralateral hemisphere were quantified utilizing ImageJ. Infarct was assessed by deducting the unaffected area of the ipsilateral hemisphere from the corresponding contralateral hemisphere area. Edema was assessed as the area difference between the ipsilateral and contralateral hemispheres. The distance between brain slices was denoted by *H*, whereas the infarct or edema region of two contiguous brain slices was represented by *S*_*n*_, *S*_*n* + 1_. The calculation formula for brain infarct and edema volume is as follows:

$$\begin{array}{c} V=\sum_{1}^{n}\frac{H\times \left(S_{n}+S_{n+1}+\sqrt{S_{n}\times S_{n+1}}\right)}{3} \end{array}$$

### 2.3 Neurobehavioral Assessment

The modified neurological severity scores (mNSS) served as a comprehensive indicator of the neurological function of the mice, with lower scores indicating better motor function and neurological recovery. Neurobehavioral testing was performed at 1, 2, and 3 days of tMCAO following previously described methods [21]. Behavioral testing and documentation were undertaken by an investigator with no knowledge of the study details to ensure objective evaluation. The mNSS score ranges from 0 to 12 and is composed of two components: (1) motor function test (0–6), which includes lifting the mouse from the tail (0–3) and observation of mice crawling state (0–3), and (2) beam walking balance test (0–6).

### 2.4 Immunofluorescence Labeling

Brain slices were preserved in 4% (w/v) paraformaldehyde (PFA; Sinopharm Chemical Reagent Co. Ltd., Shanghai, China) and 0.3% (v/v) Triton X (Sigma, MO, USA). Sections were subsequently incubated with bovine serum albumin (Meilunbio), followed by exposure to primary antibodies against NeuN (rabbit, 1:200, abn78; Millipore) and TNF‐α (rabbit, 1:200, ab183218; Abcam). After rinsing, secondary antibodies from Invitrogen (CA, USA) were applied. Apoptosis in brain tissue was examined using a TUNEL assay. The frozen brain slices were exposed to the working reagent of TUNEL Assay Kit (Meilunbio, Dalian). The sections were sealed for subsequent investigation. The total count of positive cells was determined utilizing ImageJ.

### 2.5 Western Blot and Real‐Time PCR (RT‐PCR) Analyses

The striatum was then dissected from the brain sections and placed in a RIPA protein lysis buffer. Subsequently, the striatum was subjected to ultrasonication to accelerate protein lysis. Following centrifugation, the supernatant was harvested for later western blot, and RNA was isolated with prechilled TRIzol (Invitrogen, OH).

The experiments were conducted according to procedures reported in prior studies [22]. A BCA quantification kit (Meilunbio, Dalian) was employed to measure protein concentration. Proteins were denatured by incubating at 95°C for 5 min and then loaded onto the SDS‐PAGE gel. The PVDF membranes were washed and subsequently blocked with a blocking buffer (Epizyme, China) for 15 min. Thereafter, membranes were treated with primary antibodies (Table S1) for 16 h. After exposure to secondary antibodies, the chemiluminescence signals were detected using the working solution of the chemiluminescence kit (Epizyme). Resulting chemiluminescence image quantification was conducted with ImageJ.

The RT‐PCR procedure was performed according to previously reported methods [23]. The isolated RNA was processed for cDNA synthesis with a kit obtained from Yeasen Biotech according to the prescribed parameters. For RT‐PCR, a 7900HT (ABI, Foser City, CA) real‐time PCR detecting system was used along with a qPCR SYBR Mix (Yeasen, China) following the recommended protocols. The primer sequences utilized for the PCR are enumerated in Table S2. Data quantification was conducted using the comparative CT method (2^−ΔΔCT^).

### 2.6 Ethics Statement

All experimental procedures involving animals were approved by the Institutional Animal Care and Use Committee (IACUC) of Shanghai Jiao Tong University (Approval No. 2022008) and were performed in accordance with ethical principles. The reporting of the in vivo experiments complied with the ARRIVE Guidelines.

### 2.7 Statistical Analysis

The investigation utilized GraphPad Prism 9 software for data processing. All data in the article were reported as means ± standard deviations (SDs). All data underwent the Shapiro–Wilk normality test, and nonparametric tests were employed for data that deviated from a normal distribution. Pairwise comparisons were performed using the *t*‐test, whereas comparisons across multiple groups were executed via one‐way or two‐way ANOVA. When ANOVA revealed significance, Dunnett's, Tukey's, or Šídák's post hoc test was used as appropriate. A *p* value of < 0.05 was considered statistically significant.

## 3 Results

### 3.1 Apoptosis and Necroptosis Activation Following tMCAO

To investigate the dynamic progression of RIPK1‐related pathological changes and to determine the time window in which GFH312 is most likely to exert its maximal effect, we assessed apoptosis and necroptosis activation at 2, 6, and 12 h and 1, 3, and 7 days after tMCAO. Immunofluorescence staining revealed that the proportion of NeuN^+^/TUNEL^+^ apoptotic neurons increased gradually from 2 h and reached a plateau at 1–3 days, with almost no apoptotic neurons at 7 days (Figure 1a,b). Meanwhile, western blot analysis revealed that the necroptosis‐related proteins RIPK1, RIPK3, and MLKL were upregulated after ischemic injury, with peak expression observed at 3 days (Figure 1c,d). Among the phosphorylated proteins, P‐RIPK1 and P‐RIPK3 showed an increasing trend over time after stroke, although these changes lacked statistical significance, whereas P‐MLKL was significantly elevated at 3 days and decreased at 7 days (Figure 1c,d). Collectively, these data indicate that after stroke, apoptosis‐ and necroptosis‐related pathways are activated to varying degrees and become more pronounced over time, with the most substantial alterations happening during the acute poststroke phase, especially on Day 3. These findings further imply that, as a RIPK1 inhibitor, GFH312 may exert its maximal therapeutic effect when administered during the acute phase after stroke.

**FIGURE 1:**
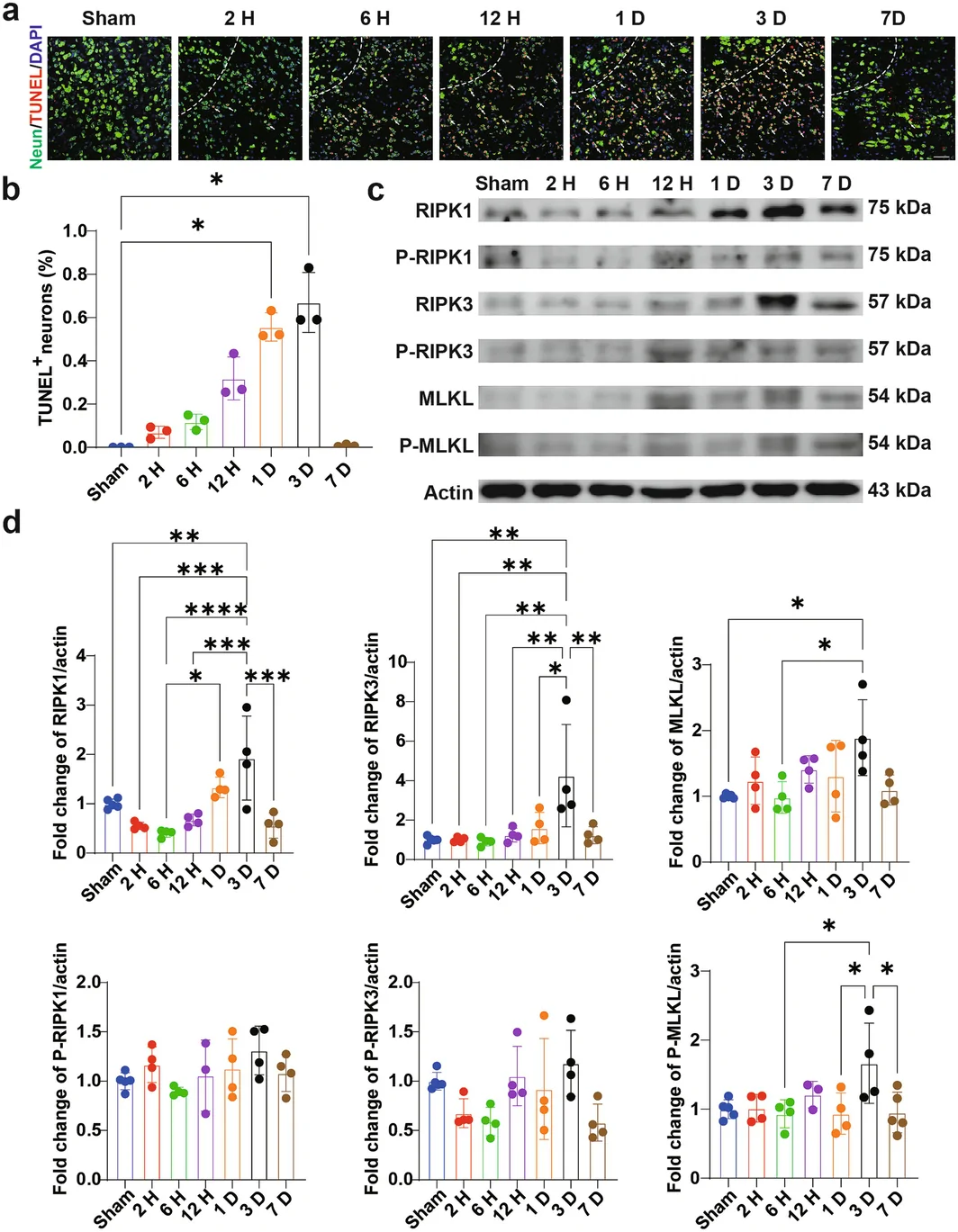
The initiation of apoptosis and necroptosis is time‐varying following tMCAO. (a) Immunostaining of apoptotic neurons labeled for TUNEL (red) and NeuN (green) in the peri‐lesion area at 2, 6, 12 h, and 1, 3, and 7 days after MCAO. Arrows indicating TUNEL^+^/NeuN^+^ cells. The dotted lines outline the border between the infarct area and the healthy area. Scale bars = 50 μm. (b) The bar graphs show the ratio of TUNEL^+^ neurons in the perifocal region at different time points of tMCAO. Statistical analysis was derived from nine slices per group, *n* = 3 mice per group. One‐way ANOVA. (c) Representative immunoblots of necroptosis‐related protein at 2, 6, 12 h, and 1, 3, 7 days after MCAO. (d) Quantification of protein expression level. *n* = 3–5 mice. Differences were evaluated by one‐way ANOVA. *p < 0.05, **p < 0.01, ***p < 0.001, ****p < 0.0001.

### 3.2 GFH312 Reduced Cerebral Infarct Volume and Facilitated Neurobehavioral Recovery

To elucidate the role of a RIPK1 inhibitor GFH312 in ischemic stroke, the entire experiment was carried out based on the experimental design shown in Figure 2a. Our findings revealed that 3 days after tMCAO, GFH312 significantly reduced the infarct volumes and edema volumes in the brain tissues when relative to the vehicle group (Figure 2b,c). Consistent with these histological findings, GFH312‐treated mice showed considerably improved neurological function, as evidenced by lower mNSS scores at 3 days after tMCAO relative to the vehicle group (Figure 2d). Moreover, GFH312 attenuated poststroke body weight loss (Figure 2e). Collectively, these results demonstrate that GFH312 alleviates brain injury and promotes the restoration in ischemic mice during the acute poststroke phase.

**FIGURE 2:**
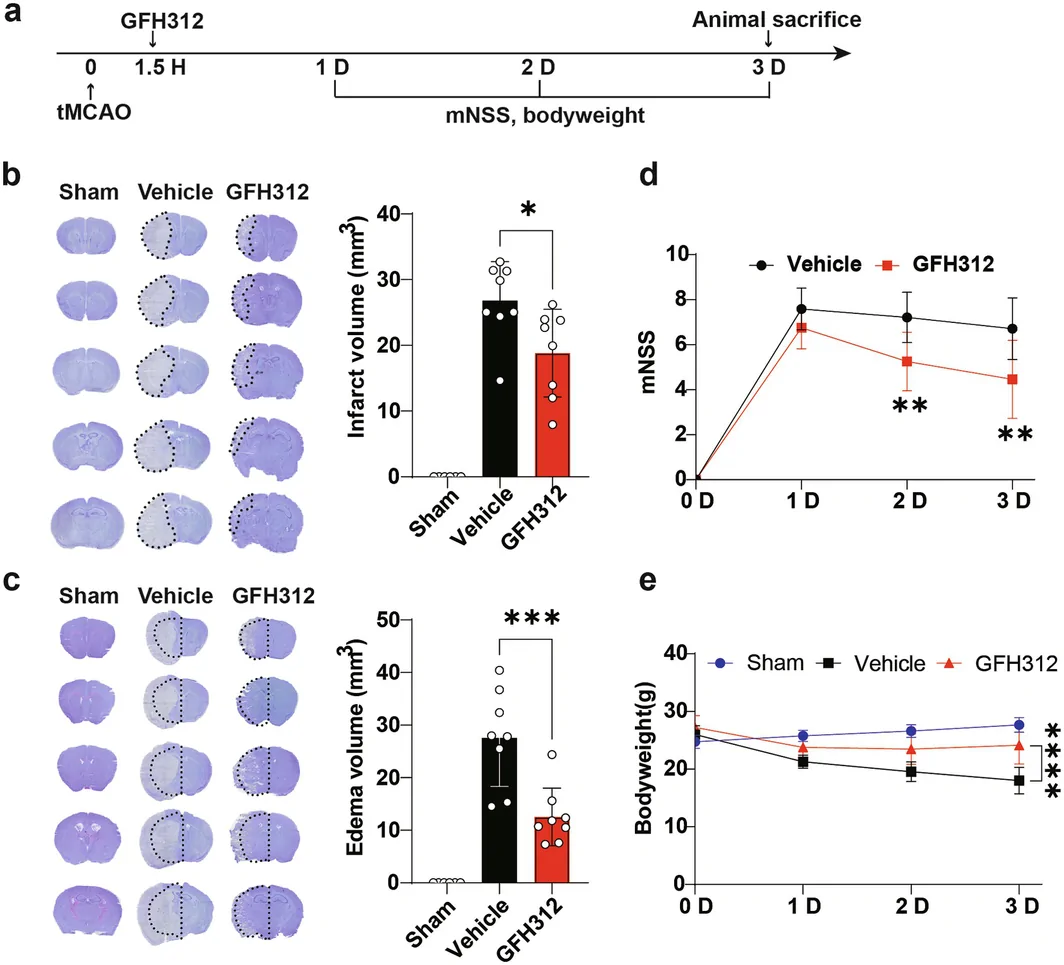
GFH312 reduced brain infarct volume and promoted neurobehavioral recovery during the acute poststroke phase. (a) Schematic representation of the experimental design with animals. (b) Representative images of cresyl violet‐stained brain slices at 3 days posttreatment. The infarct region of the mouse brain was delineated by a dashed line, and the bar graph (right) presented a quantitative comparison of the infarct volume. *n* = 6–8 mice per group. One‐way ANOVA. (c) The dashed line delineated the contralateral hemisphere of the mouse brain in the cresyl violet‐stained slices. Bar graph shows the quantification of the edema volume. *N* = 6–8 mice. Analysis using one‐way ANOVA. (d) The neurobehavioral recovery was assessed by mNSS. *n* = 12 mice. Two‐way ANOVA. (e) Bodyweight of three group mice was recorded each day after ischemic stroke. *n* = 12 mice per group. Two‐way ANOVA. *p < 0.05, **p < 0.01, ***p < 0.001, ****p < 0.0001.

### 3.3 GFH312 Inhibited Neuronal Apoptosis and Necroptosis‐Related Pathways

To investigate the mechanisms behind the neuroprotective effects of GFH312 following tMCAO, we next examined whether its beneficial effects were accompanied by alterations in apoptosis and RIPK1‐dependent necroptotic signaling in stroke mice. As shown by immunofluorescence analysis, a substantial quantity of neurons underwent apoptosis in the peri‐infarct area of the vehicle group, while the proportion of NeuN^+^/TUNEL^+^ apoptotic neurons was significantly decreased after treatment with GFH312 (Figure 3a,b). Additionally, the western blotting results indicated a considerable downregulation in the cleaved Caspase‐3 abundance and the cleaved Caspase‐3/Caspase‐3 ratio following GFH312 treatment, suggesting a suppression of apoptotic activation (Figure 3c,d).

**FIGURE 3:**
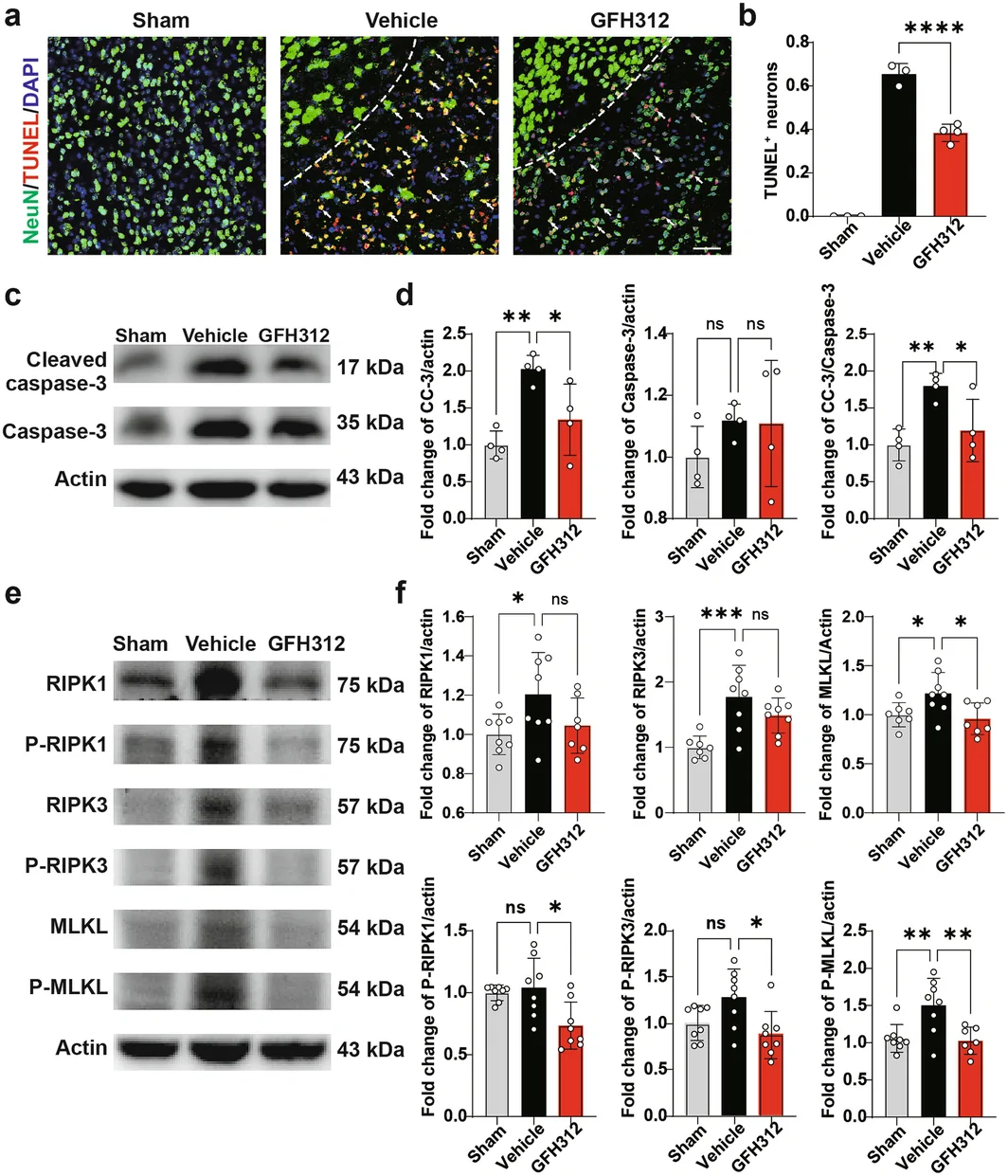
GFH312 inhibited neuronal apoptosis and necroptosis‐related pathway after tMCAO in mice. (a) Immunostaining of apoptotic neurons labeled for TUNEL (red) and NeuN (green) in the mouse brain at 3 days poststroke. Arrows indicating TUNEL^+^/NeuN^+^ cells. The dotted lines outline the border between the infarct area and the other area. Scale bar = 50 μm. (b) The graph depicts the fraction of neurons positive for TUNEL staining in the peri‐lesion zone after tMCAO. *n* = 3–4 mice. Analysis using one‐way ANOVA. (c) Immunoblots of caspase‐3 and cleaved caspase‐3 in the brain of the GFH312‐treated and vehicle mice at 3 days of tMCAO. (d) Quantification of protein expression in b. *N* = 4 mice. Analysis using one‐way ANOVA. (e) Immunoblots of RIPK1, P‐RIPK1, RIPK3, P‐RIPK3, MLKL, and P‐MLKL in the mouse brain at 3 days of tMCAO. (f) Quantification of expression level of protein in e. *n* = 7–8 mice. Analysis using one‐way ANOVA. *p < 0.05, **p < 0.01, ***p < 0.001, ****p < 0.0001. ns, not significant.

In addition to apoptosis, we further examined whether GFH312 modulates another major RIPK1‐associated cell death pathway—necroptosis. Western blot analysis showed that, compared with the vehicle group, GFH312 treatment substantially reduced protein levels of MLKL, phosphorylated RIPK1, phosphorylated RIPK3, and phosphorylated MLKL in brain tissue, whereas total RIPK1 and RIPK3 protein levels also showed a downward trend that did not reach statistical significance (Figure 3e,f). Further analysis revealed that although phosphorylated‐to‐total protein ratios were not markedly altered (Figure S1), the absolute levels of the phosphorylated proteins were markedly decreased. Overall, these data indicate that GFH312, as a RIPK1 inhibitor, not only reduces neuronal apoptosis but may also provide neuroprotection by inhibiting RIPK1–RIPK3–MLKL‐mediated necroptotic signaling.

### 3.4 GFH312 Suppressed Neuroinflammation Following tMCAO

Following stroke, necrotic cell‐derived damage‐associated molecular patterns (DAMPs), in conjunction with RIPK1 activation, might exacerbate neuroinflammatory responses and hence deteriorate stroke outcomes. Considering the pivotal function of RIPK1 in modulating cell fate and inflammatory signaling, we subsequently assessed the impact of GFH312 on acute neuroinflammation following tMCAO. Immunofluorescence labeling revealed an impressive rise in TNF‐α^+^ cells in the brains of vehicle‐treated animals following tMCAO, whereas GFH312 treatment considerably decreased the number of TNF‐α^+^ cells (Figure 4a,b). The mRNA abundance of TNF‐α, IL‐1α, IL‐1β, IL‐6, and IL‐17 was substantially higher in the brains of vehicle‐treated animals versus the sham group, and was notably reduced by GFH312 treatment (Figure 4c). The results demonstrate that GFH312 significantly mitigates immediate neuroinflammatory reactions following tMCAO.

**FIGURE 4:**
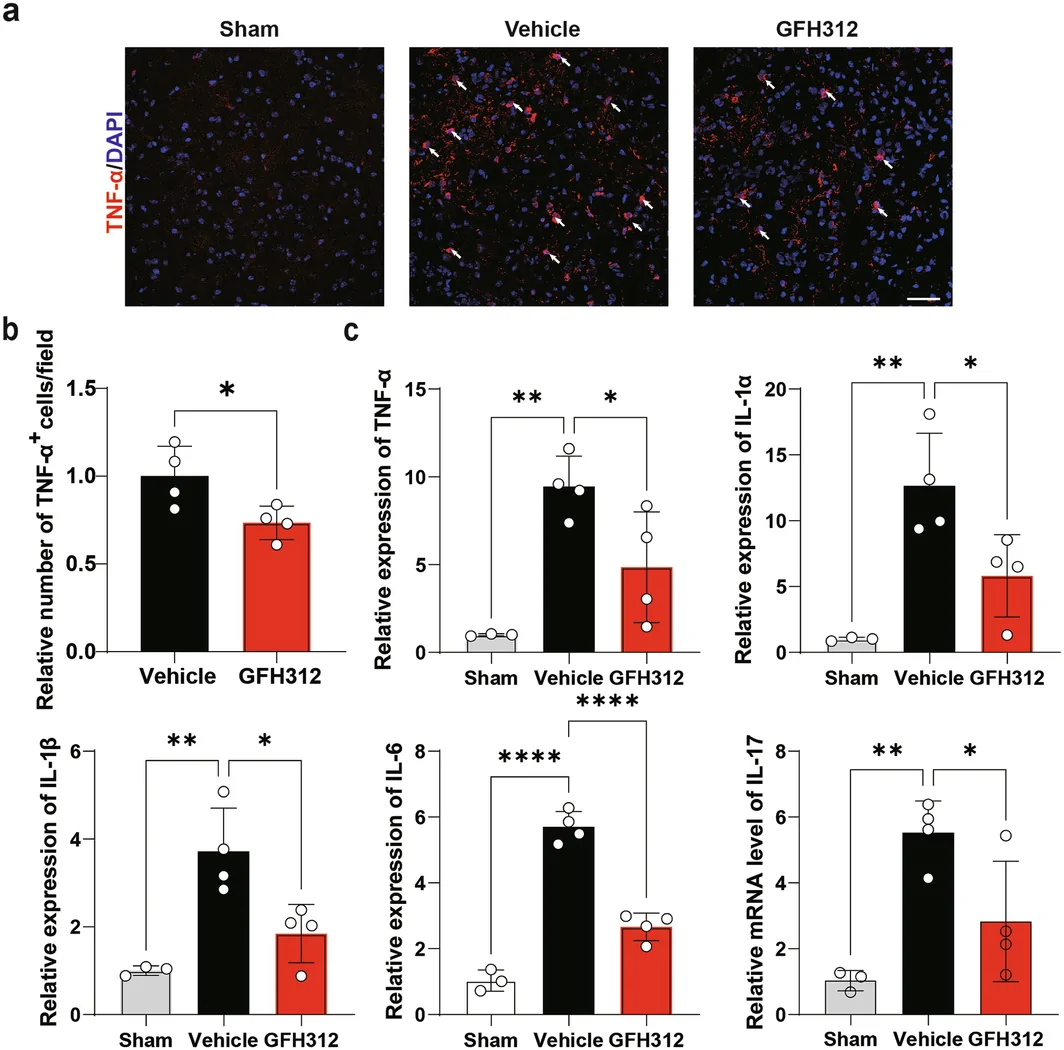
GFH312 suppressed neuroinflammation after ischemia in mice. (a) Representative immunostaining of TNF‐α^+^ cells in mouse brain at 3 days after MCAO. Arrows indicating TNF^+^ cells. Scale bars = 50 μm. (b) Quantification of TNF‐α^+^ cells at 3 days of ischemic stroke. *n* = 4 mice. Data from the vehicle group were normalized to 1. Unpaired Student's *t*‐tests. (c) Real‐time PCR analysis of inflammatory factor expression in the mouse brain at 3 days following MCAO. *n* = 3–4 mice. GAPDH served as an internal control. Data from the sham group were normalized to 1. Analysis using one‐way ANOVA. *p < 0.05, **p < 0.01, ****p < 0.0001.

## 4 Discussion

In the current study, we investigated the temporal occurrence of apoptosis and necroptosis poststroke, and confirmed the beneficial effects of the RIPK1 inhibitor GFH312 in ischemic stroke as well as its potential mechanisms. We found that apoptosis and RIPK1‐dependent necroptosis occurred rapidly after stroke onset and peaked during the acute phase. Continuous administration of GFH312 during the acute poststroke phase exerted neuroprotective effects in stroke mice, effectively reducing brain injury and improving neurological behavioral performance. Furthermore, at the mechanistic level, as a RIPK1 inhibitor, GFH312 indeed alleviated neuronal apoptosis and the severity of necroptosis in brain tissue by inhibiting RIPK1, thereby diminishing the expression of several neuroinflammation‐associated genes and proteins.

To determine our treatment window and the in vivo occurrence of the two forms of RIPK1‐mediated cell death, this study first systematically observed the dynamic changes of apoptosis and necroptosis at different time points after ischemic stroke. TUNEL^+^ cells can serve as significant indicators of apoptosis [24], and alterations in the phosphorylation levels of RIPK1, RIPK3, and MLKL imply the potential involvement of necroptosis in poststroke brain damage progression [25]. The modifications in the number of TUNEL^+^ neurons after stroke indicated that apoptosis gradually increased during the acute phase after stroke and peaked on Day 3, which is consistent with the previous view that cell apoptosis mainly occurs during the acute early phase [11, 26]. We observed that the total protein levels of RIPK1, RIPK3, and MLKL exhibited a time‐dependent rise following stroke and reached relatively high levels at 1–3 days, suggesting that the RIPK1–RIPK3–MLKL‐related signaling pathway was continuously mobilized after ischemic brain injury. Notably, on the third day after stroke, the proportion of phosphorylated proteins relative to total proteins did not change significantly (Figure S1), which may be due to the marked increase in total RIPK1 and RIPK3 protein expression after stroke, affecting the changes in phosphorylation ratios. Moreover, although P‐RIPK1 and P‐RIPK3 did not show significant changes, P‐MLKL was markedly increased 3 days after stroke. Given that MLKL phosphorylation is considered a key molecular event in the execution phase of necroptosis, this also suggests that necroptosis remains involved in the brain injury process during the early phase after stroke.

After clarifying the dynamic changes of RIPK1‐involved cell death, we set the timing of GFH312 treatment in the acute phase because by the subacute phase, cell death may no longer be the dominant process driving stroke progression. After 3 days of treatment, the recovery of tissue injury, neurological behavior, and body weight in stroke mice suggested that GFH312 has a definite protective effect during the acute poststroke phase. RIPK1 is a key node in the regulation of cell death and inflammatory signaling [27]; therefore, pharmacological inhibition of RIPK1 may simultaneously interfere with multiple injury‐related pathways, thereby producing a more comprehensive neuroprotective effect as reflected by the inhibitory effects of GFH312 on apoptosis and necroptosis observed in our study. As a newly developed RIPK1 inhibitor, the effectiveness of GFH312 in this study further reinforces the view that RIPK1 could serve as an attractive candidate for stroke treatment.

In addition to regulating cell death, GFH312 also showed a marked anti‐inflammatory effect. The expression and release of large numbers of inflammatory factors from different cellular sources after stroke, such as TNF‐α [28], can activate RIPK1 signaling [29], and this signaling can in turn further amplify inflammation and promote the release of more pro‐inflammatory factors, forming a vicious cycle, the ultimate consequence of which is more cell death [30]. This study found that GFH312 significantly lowered the quantity of TNF‐α^+^ cells and suppressed the expression of inflammatory mediators, indicating its potential to effectively mitigate the acute neuroinflammatory response following tMCAO and interrupt this detrimental cycle. Considering that there is a mutually promoting relationship between inflammation and cell death, the inhibitory effect of GFH312 on inflammation may also be an important component of its neuroprotective action.

This study still has some limitations. First, this study mainly focused on the acute phase after stroke, and the long‐term effects of GFH312 remain to be further clarified in future work. Second, although our data consistently support the involvement of RIPK1‐related signaling in the neuroprotective effects of GFH312, the current study did not include additional target‐validation approaches such as RIPK1 genetic knockdown/knockout or complementary pharmacological inhibitors. Therefore, our findings should be interpreted as supporting a RIPK1‐related mechanism rather than establishing definitive target specificity in ischemic brain tissue. Third, because the present analyses were performed mainly at the tissue level, the cell type‐specific effects of GFH312 on neurons, microglia, astrocytes, and other brain cell populations remain to be further clarified. Future studies incorporating genetic validation, complementary RIPK1 inhibitors, and cell‐specific approaches will help define the precise molecular and cellular mechanisms underlying the protective effects of GFH312 after ischemic stroke.

## 5 Conclusion

This work illustrates that the new RIPK1 inhibitor GFH312 exhibits substantial neuroprotective benefits following ischemic stroke and enhances stroke outcomes. Subsequent analysis revealed that GFH312 can suppress neuronal apoptosis, disrupt the RIPK1‐associated necroptosis signaling cascade, and significantly mitigate the acute neuroinflammatory response. The findings indicate that RIPK1 could serve as a significant therapeutic target for ischemic stroke, and that GFH312 may emerge as a possible intervention therapy that concurrently modulates cell death and inflammatory responses.

## Author Contributions

G.‐Y.Y. originated the research. Z.L. completed the experimental work, conducted the data analysis, and drafted the paper. S.W., Q.S., C.L., R.S., J.Y., and H.K. facilitated experimental procedures. Y.T., Z.Z., and W.L. contributed to the design of experiments. G.‐Y.Y., J.‐Y.Z., and F.‐S.Z. (GenFleet Therapeutics (Shanghai) Inc.) rendered financial assistance. All contributors provided commentary on and/or revised the paper.

## Funding

This work was supported by GenFleet Therapeutics (Shanghai) Inc (project number: GF‐107).

## Conflicts of Interest

The authors declare no conflicts of interest.

## Supporting information


**Figure S1:** Quantification of the ratios of phosphorylated to total necroptosis‐related proteins after tMCAO.
**Table S1:** Primary antibody information.
**Table S2:** Mouse primer sequences.

## Data Availability

The data that support the findings of this study are available from the corresponding author upon reasonable request.
