# Supplementary material for: GFH312 Reduces Neuronal Death by Inhibiting RIPK1‐Mediated Apoptosis and Necroptosis After Ischemic Stroke
Source: CNS Neurosci Ther. 2026 Oct 7;32(10):e71053. doi: 10.1002/cns.71053 (PMC13644914; doi:10.1002/cns.71053)
Supplement: Supplementary file 1 [file CNS-32-e71053-s001.docx]

**Supplementary Figure 1**

**Supplementary Figure 1. Quantification of the ratios of phosphorylated to total necroptosis-related proteins after tMCAO.**

n = 6–8 mice. Analysis using one-way ANOVA.

**Supplementary table 1. Primary antibody information.**

| **Antibody** | **Product code** | **company** | **dilution ratio** |
| --- | --- | --- | --- |
| Caspase-3 | 9662 | CST | 1:1000 |
| Cleaved Caspase-3 | 9661 | CST | 1:1000 |
| RIPK1 | 3493 | CST | 1:1000 |
| RIPK3 | 95702 | CST | 1:1000 |
| MLKL | 37705 | CST | 1:800 |
| P-RIPK1 | 31122 | CST | 1:1000 |
| P-RIPK3 | 91702 | CST | 1:800 |
| P-MLKL | 37333 | CST | 1:800 |
| β-actin | 66009 | Proteintech | 1:5000 |

**Supplementary table 2. Mouse primer sequences.**

| **Genes** | **Forward primer** | **Reverse primer** |
| --- | --- | --- |
| *Tnfa* | TAGCCAGGAGGGAGAACAGA | CCAGTGAGTGAAAGGGACAGA |
| *IL-1α* | CGCTTGAGTCGGCAAAGAAAT | CTTCCCGTTGCTTGACGTTG |
| *IL-1β* | TACATCAGCACCTCACAAGC | AGAAACAGTCCAGCCCATACT |
| *IL-6* | ACCAAGACCATCCAATTCATC | CTGACCACAGTGAGGAATGTC |
| *IL-17* | GTTCGTGCTATTGATTTTCAGC | GGACCCCTTTACACCTTCTTT |
| *GAPHD* | AAATGGTGAAGGTCGGTGTG | AGGTCAATGAAGGGGTCGTT |
